# Neural circuits driving larval locomotion in *Drosophila*

**DOI:** 10.1186/s13064-018-0103-z

**Published:** 2018-04-19

**Authors:** Matthew Q. Clark, Aref Arzan Zarin, Arnaldo Carreira-Rosario, Chris Q. Doe

**Affiliations:** 1 Institute of Neuroscience, Institute of Molecular Biology, Howard Hughes Medical Institute, University of Oregon, Eugene, OR 97403 USA; 2 Division of Biology and Biological Engineering, California Institute of Technology, Pasedena, CA 91125 USA

**Keywords:** Locomotion, Locomotor circuits, Sensorimotor, Wave propagation, Navigation, Neurodevelopment, Multisensory integration

## Abstract

More than 30 years of studies into *Drosophila melanogaster* neurogenesis have revealed fundamental insights into our understanding of axon guidance mechanisms, neural differentiation, and early cell fate decisions. What is less understood is how a group of neurons from disparate anterior-posterior axial positions, lineages and developmental periods of neurogenesis coalesce to form a functional circuit. Using neurogenetic techniques developed in *Drosophila* it is now possible to study the neural substrates of behavior at single cell resolution. New mapping tools described in this review, allow researchers to chart neural connectivity to better understand how an anatomically simple organism performs complex behaviors.

## Background

Our central nervous system (CNS) is composed of billions of neurons with orders of magnitude more synaptic connections that form the basis of neural circuits that produce complex behaviors. Challenges faced by twenty-first century neuroscientists, as articulated by the BRAIN initiative, include characterizing neuronal diversity, making maps at various scales, observing the brain in action, and demonstrating causality among anatomical circuit elements [1]. All of these goals are rapidly being realized in the study of *Drosophila* locomotor circuits, which can provide a model for characterizing larger nervous systems.

**Fig. 1:**
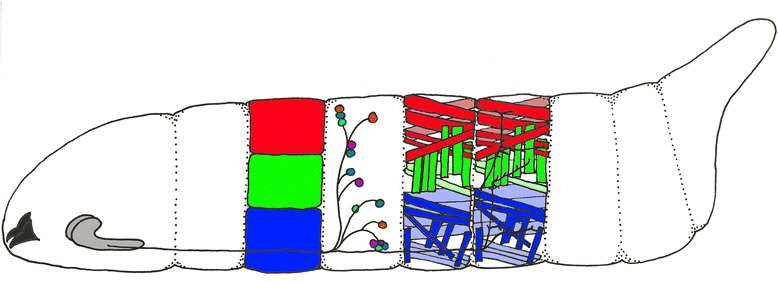
Muscles and motor neurons that drive various locomotor behaviors. Schematic of Drosophila larva side view, anterior to left. Mouthhooks far left, black; CNS with anterior brain lobes and ventral nerve cord, grey. Nerves contain sensory input from abdominal segments (small circles) and motor neuron output to muscles (red/green/blue rectangles). The red/green/blue territories represent muscle functional groups containing ~ 10 individual muscles each: red is dorsal longitudinal muscles, green is transverse muscles, and blue is ventral longitudinal muscles. Some of these individual muscles are shown in the same color code in more posterior segments. This larva shows only seven segments for clarity; wild type larvae contain three thoracic segments and eight abdominal segments

Patterned motor behaviors such as locomotion require the coordination of neural circuits which is accomplished by central pattern generators (CPGs) [2]. CPGs are microcircuits comprised of excitatory and inhibitory neurons. The net activity of CPGs can be observed at the level of rhythmic activity in muscles or motor neurons. Much of our understanding of the origins of motor pattern generation is from the study of invertebrates such as crabs, crayfish, lobsters, leech and locusts [3–6]. Owing to their small size, complex neural circuits in *Drosophila* have traditionally proven difficult to study. However, recent developments have allowed in-depth analysis of neural circuits and behavior: new tools provide genetic access to single neurons [7, 8], the ability to monitor activity or activate/silence neurons (Table 1), perform trans-synaptic tracing [9, 10], and most importantly the completion of a serial section transmission electron microscopy (TEM) reconstruction of the entire larval CNS [11–15]*. Drosophila* larvae have stereotyped anatomy (Fig. 1), behaviors, anatomical simplicity, genetic accessibility, and transparent cuticle, which allows for live-imaging of neuronal activity during crawling behaviors [16, 17]. It is a time of rapid progress, and we summarize studies of *Drosophila* larval locomotion as of January 2018.

**Table 1:** Tools for neural circuit analysis

(1) Binary expression systems. There are three driver/reporter systems commonly used in Drosophila: Gal4-UAS, LexA-lexAOP, and QF-QUAS [87–89]. Over 8000 cis-regulatory module (CRM)-Gal4 lines have been generated with many expressed in fewer than 10 neurons per brain lobe or fewer than 5 neurons per VNC hemisegment [7, 8]. These lines are modular so that the CRMs can be easily swapped to drive split-Gal4 elements or the Gal4 repressor Gal80, allowing intersectional expression patterns as sparse as single neurons [64, 90–93]. In addition, the CRMs can be used to drive other binary driver elements (LexA or QF), allowing two different genes to be expressed in two different neurons (e.g. Gal4/UAS to drive the neuronal activator Chrimson in putative input neurons, and LexA/LexAOP to drive the neural activity sensor GCaMP in putative downstream target neurons). (2) Neuronal activators and silencers. Commonly used neuronal activators are red-light activated Chrimson or ReaChR; green light activated channelrhodopsin (ChR); or warmth activated TrpA1 [94–96]. Neuronal silencers include the constitutively active inwardly rectifying potassium channel KiR [97] and light chain of tetanus toxin (TNT) [98], the yellow light-activated Halorhodopsin [25], the recently developed blue light activated anion channelrhodopsin GtACR [99, 100], or temperature-sensitive Shibire [101]. (3) Activity monitors. Genetically encoded calcium indicators (GECIs) Neuronal activity monitors include the GCaMP6 series (fast, medium, slow) and the more recent red-shifted RCaMP and RGECO [102].

### Natural crawling behaviors

**Fig. 2:**
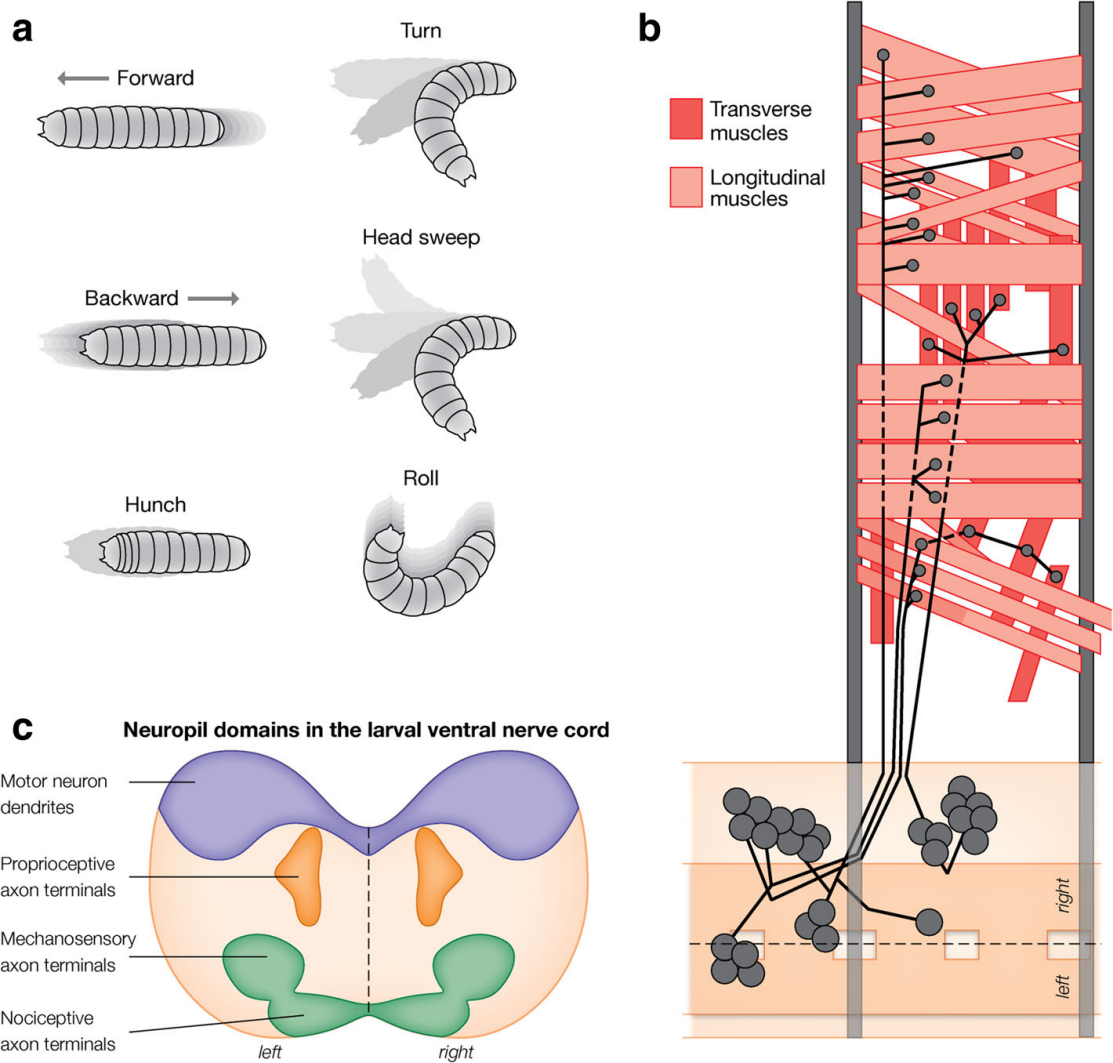
Muscles and motor neurons that drive various locomotor behaviors. **a** Larval locomotor behaviors. **b** Abdominal motor neurons and muscles in a single hemisegment. Only the type Ib motor neurons are shown (big bouton/single muscle target). Longitudinal muscles are light red, transverse muscles are darker red. Anterior to left; ventral midline, dashed line; dorsal midline at top of panel. **c** Cross-section schematic of abdominal neuropil; surrounding cell bodies are not shown. Motor dendrites target the dorsal (most internal) domain, sensory axons target ventral (most superficial) domains, with the exception of proprioceptive axons that target an intermediate domain. Ventral midline separating left/right sides, dashed line

*Drosophila* larvae spend their lives continually foraging for food as they have a limited time to obtain a nutrient-dependent critical weight that must be met in order to undergo metamorphosis [18]. Natural crawling behaviors include turns, head sweeps, pauses, hunching, bending, burrowing, rolling (escape) and forward and backward locomotion [19–21] (Fig. 2a). Here we focus on forward and backward locomotion, which are among the best-characterized larval behaviors. Larval locomotion is generated by abdominal somatic body wall muscle contractions moving from posterior to anterior (forward locomotion) or anterior to posterior (backward locomotion) [22–25]. Consecutive bouts of forward or backward waves are called runs.

All of these complex movements are enabled by a larval body plan that is regionally specified by Hox genes. Hox genes give segmental identity and regional specification to the central brain, subesophageal zone (SEZ) and the ventral nerve cord (VNC) which includes 3 thoracic segments, 8 abdominal segments and a terminal plexus [26–28]. It is hypothesized that Hox gene networks may govern the regional specification of peristaltic locomotion circuits through modifying CPG organization [29]. For example, neural control of turning movements is located within the thoracic segments of the VNC [30] while the CPGs that drive larval locomotion reside in the thoracic and abdominal segments of the VNC [31, 32]. Additional ‘command-like’ descending neurons in the SEZ and central brain can direct locomotion behaviors [33]. However, little is known about the interneurons used in region-specific aspects of locomotion, such as forward or backward movements, head sweeps, rolling, or pauses. Identifying individual neurons participating in specific behaviors will be necessary to shed light on this question of regional specialization.

### Motor and sensory neurons are well-defined elements of the locomotor system

The larval somatic body wall muscles and motor neurons that innervate them are highly stereotyped, and responsible for driving forward and backward waves of muscle contraction [22]. In each abdominal hemisegment, there are 30 muscles arranged in two major groups: the longitudinal muscles are aligned with the body axis, whereas the transverse muscles are orthogonal to the body axis, i.e. circumferential [34] (Fig. 2b). Each body wall muscle is innervated by a single motor neuron with “big” boutons (Ib motor neurons), and the three functionally related groups of muscles (dorsal longitudinal, ventral longitudinal, and transverse) are also innervated by single motor neurons covering the group with “small” boutons (Is motor neurons) [35, 36]. In addition, three ventral unpaired midline type II motor neurons per segment release the neuromodulator octopamine [37]. Both 1b and 1 s motor neurons provide glutamatergic excitatory drive to the muscles, and several 1b motor neurons have been shown to be rhythmically active during waves of muscle contraction during forward or backward locomotion [38]; whether all 1b and 1 s motor neurons participate in forward and backward locomotion is not known. Interestingly, whole-cell patch-clamp dual recordings showed that 1b motor neurons (big boutons on a single muscle target) are more easily recruited than Is motor neurons [39], and live imaging showed that muscle contraction is most closely associated with type Ib activity [38]. It is likely that larval Ib and Is motor neurons are similar to motor neurons in crayfish or humans where low and high activation threshold motor neurons facilitate powerful or precise movements, respectively [40, 41].

Motor neuron dendritic domains form a myotopic map within the CNS neuropil, and all motor dendrites target the dorsal neuropil (Fig. 2c). Each functional class has a slightly different domain: dorsally projecting motor neurons have more lateral arbors and ventrally projecting motor neurons have more medial dendritic arbors [27, 42, 43]. This suggests that premotor neurons may select among functional pools of motor neurons by targeting their axons to specific regions of the neuropil.

There are 42 sensory neurons that bilaterally tile each hemisegment of the body wall in a modality specific array [44, 45]. Motor patterns can be generated independent of sensory input, but peristaltic muscle contraction waves are slower and locomotion is slower and less coordinated [23, 32, 46–50]. Multidendritic (md) branched neurons are among the best-characterized sensory neurons. The dendritic arborization (da) neurons mds are specialized and classified into four types (class I-IV) that vary in the degree of branching complexity with class I being the simplest and class IV the most elaborate. Class I sensory neurons act as proprioceptors and are required for normal locomotion; class II sensory neurons are poorly characterized, though there is some evidence they function as touch receptors; class III sensory neurons are touch receptors, and class IV sensory neurons are polymodal nociceptive neurons that mediate escape behaviors [50–55]. Each sensory neuron projects to a highly stereotyped region of the neuropil: with the exception of proprioceptive neurons, all terminate in the ventral neuropil [56–59] (Fig. 2c).

### Interneurons are the most common but the least characterized VNC neuronal type

**Fig. 3:**
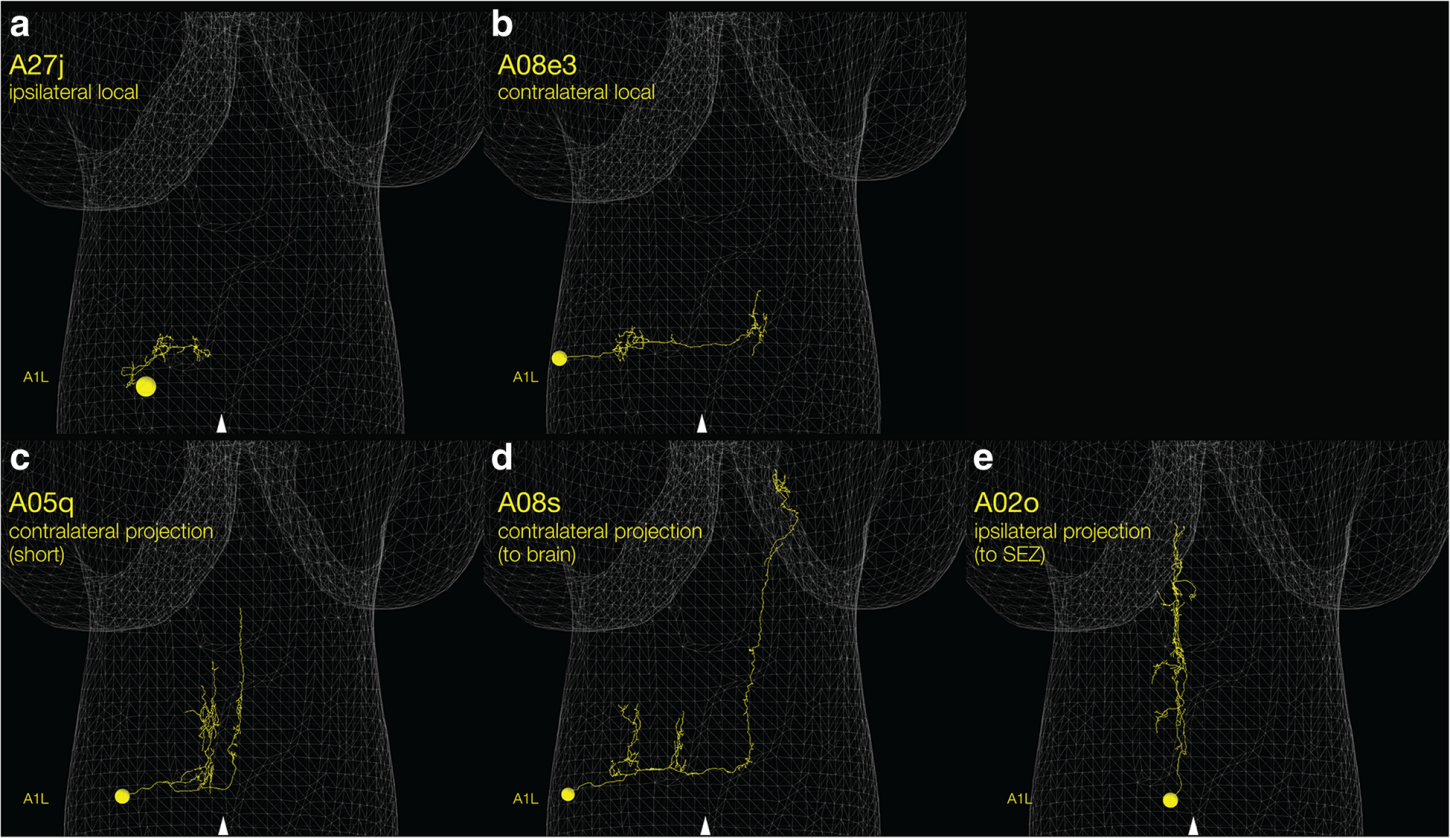
Local and projection interneurons. Examples of local and projection interneurons. There are also descending interneurons with somata in the brain, SEZ, thoracic, or upper abdominal segments (not shown). All panels show a single hemi-segment for clarity (A1 left), although the neurons are bilateral and present in more posterior abdominal segments as well. Midline, arrowhead. (**a**, **b**) Local interneurons. A27j is an ipsilateral local interneuron that confines its pre- and post-synaptic arbors to the hemisegment containing its soma [103]. A08e3 is a contralateral local interneuron that projects a process across the midline [16]. Contralateral local interneurons typically have pre-synaptic outputs contralateral to the soma, and post-synaptic inputs on ipsilateral arbors. (**c**–**e**) Projection interneurons. A05q is a contralateral projection interneuron that extends anteriorly multiple segments but does not reach the brain [85]. A08s is a contralateral projection interneuron that extends anteriorly to the brain [16]. A02o, also called the “wave” neuron, has a contralateral projection that terminates in the thorax and/or SEZ [82]. Typically, projection interneuron have pre-synaptic outputs at the anterior terminus of the ascending projection, and post-synaptic inputs on the local arbors

Whereas much is known about motor neurons and their target muscles, interneurons have been the “black box” of the *Drosophila* locomotor circuitry. Within the VNC there are ~ 250 bilateral pairs of interneurons, defined as local or projection neurons with processes staying within the CNS (Fig. 3). *Drosophila* larval interneurons are cholinergic (excitatory), GABAergic (inhibitory), or glutamatergic (inhibitory) [47, 60–64]. The role of excitatory and inhibitory interneurons in generating precisely coordinated motor activity, either within a single segment or between adjacent segments, is a rapidly advancing area of research, described below. There are also dopaminergic and serotonergic modulatory interneurons [23, 65, 66], but their role in locomotion is poorly understood. In the following sections, we will review recent studies aimed at identifying different subsets of excitatory or inhibitory interneurons in the VNC and understanding their role in controlling the intrasegmental and intersegmental motor activity during larva crawling.

### Intrasegmental coordination: interneuron inhibition generates a phase delay between distinct motor pools

Larval crawling is generated by precisely timed waves of muscle activity [32, 67–69]. These muscle contractions must be coordinated both within a segment (intrasegmental coordination), which is the topic of this section, and between segments to ensure smooth wave propagation, which is the topic of the next section.

During forward or backward locomotion, all muscles in a segment do not contract simultaneously. In both directions of locomotion, longitudinal muscles (L) start to contract before transverse muscles (T) [67], although this is followed by a phase of L and T co-contraction [70]. The partial overlapping contraction pattern of L and T muscles during larval crawling make this behavior distinct from the well-studied antagonistic muscle contraction patterns seen in left-right alternating limbs or extensor-flexor muscles in vertebrate animals [71]. In the future, it would be informative to know the timing and amplitude of each of the 30 muscles during forward, backward, and rolling locomotion.

**Fig. 4:**
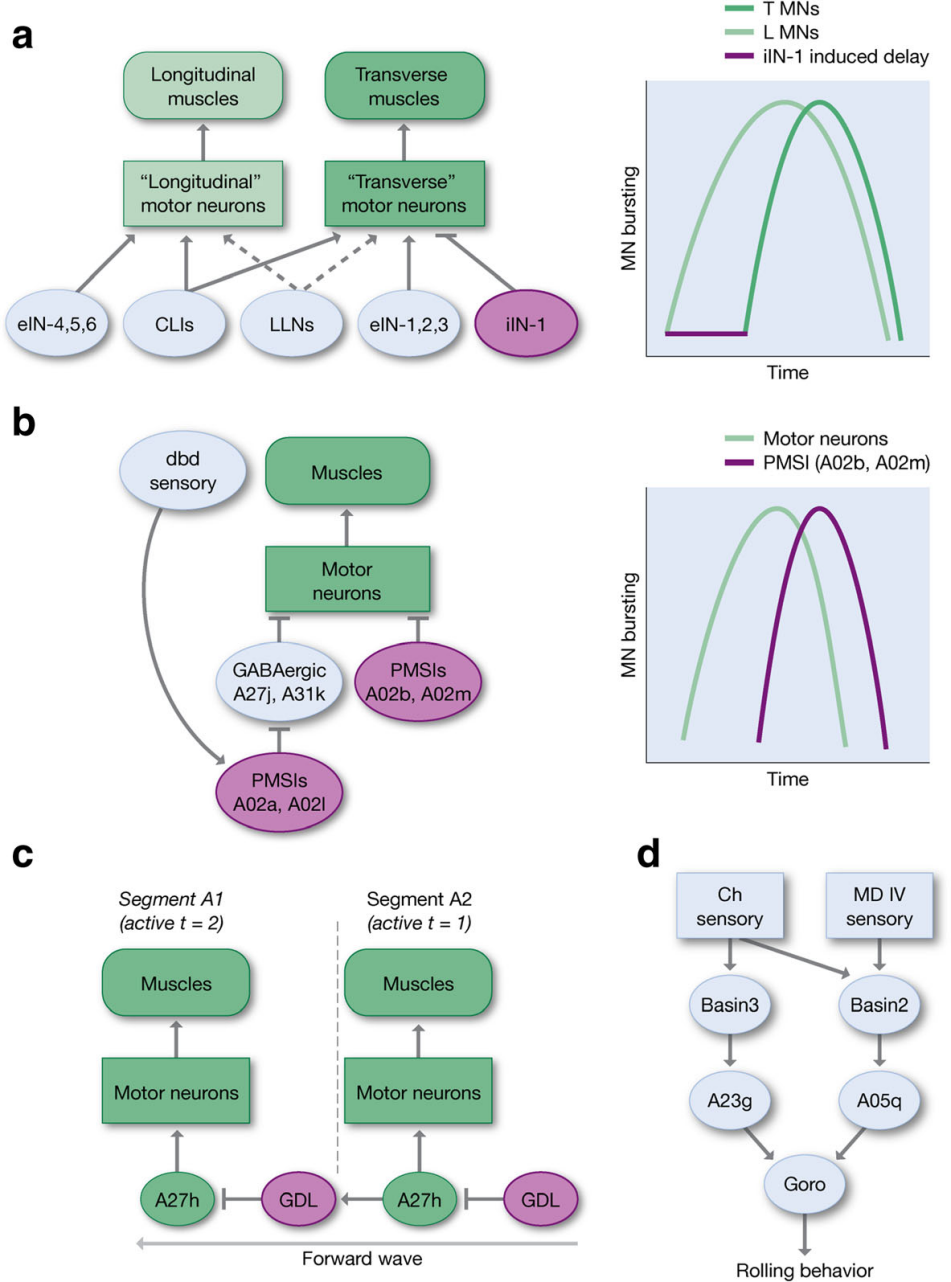
Circuit motifs used in larval locomotion. **a** Circuits leading to sequential longitudinal/transverse muscle contraction. Motor neurons innervating both longitudinal and transverse muscle groups (“longitudinal” and “transverse” motor neurons, respectively) receive similar excitatory premotor input, but the motor neurons specifically innervating transverse muscles also receive inhibitory input which leads to a delay in the initiation of transverse muscle contraction. **b** Circuits that limit the length of motor neuron activity. The PMSI A02b/A02m inhibitory premotor neurons limit the length of motor neuron firing. GABAergic A27j/A31k may also perform this function based on their neurotransmitter and connectivity, but have not yet been functionally characterized. Dbd sensory neurons are thought to be stretch receptors [104], hence activated by muscle relaxation in the segment they are tiling and/or by muscle contraction in the adjacent segments. If so, it is likely that A02a and A02l fire after A02b/A02m and A27j/A31k premotor neurons to remove the inhibition from motor neurons after their target muscles are relaxed, preparing them for the next round of firing. **c** Circuits that promote smooth progression of the muscle contraction wave during forward locomotion. The A27h premotor neuron activates motor neuron firing in a segment, while also activating the inhibitor GDL neuron in the next most anterior segment, which leads to a delay in motor activity necessary for smooth wave progression. **d** Circuits that promote larval rolling. Only the local VNC circuit is shown for clarity. Sensory input leads to activation of the Goro “command-like” neuron that is necessary and sufficient for rolling behavior

How is the L-T muscle contraction phase delay generated? The motor neurons innervating the L and T muscles show the same phase delay in fictive forward and backward behavior (isolated brains lacking sensory input) [32], indicating that the mechanisms generating this phase relationship are hard-wired within the VNC and independent of sensory feedback. The phase delay could be due to differences in intrinsic properties of T and L motor neurons, or due to differences in premotor input between L and T motor neurons. Zwart et al. (2016) did not observe any difference in the intrinsic firing properties of L or T motor neurons, ruling out the first hypothesis. To look for differences in premotor input, they used the TEM reconstruction of the larval CNS, and traced four motor neurons innervating the transverse muscles (LT1-LT4) and MN5-Ib synapsing onto a longitudinal muscle (LO1). Next they traced all the premotor neurons directly connecting to these five motor neurons. Strikingly, they identified a single GABAergic premotor neuron (iIN-1) which provides inhibitory input exclusively to LT1-LT4 motor neurons, which could introduce a delay between L and T motor neuron firing [70]. Blocking the activity of iIN-1 neuron resulted in synchronous contraction onset in the L and T muscles. Of course, this does not rule out differences in excitatory input, as well. Zwart et al. identified three excitatory premotor neurons (named eIN-1, eIN-2 and eIN-3) that innervate T but not L motor neurons, and showed that they fire synchronously with the aCC motor neuron innervating an L muscle [70]. The authors propose that inhibitory premotor input sculpts the phase delay between L and T motor firing, leading to sequential L-T muscle contraction activity within each segment during locomotion [70] (Fig. 4a). The functional relevance of the L-T phase delay is unknown.

Another aspect of generating the proper intrasegmental muscle contraction pattern is regulating the duration of motor neuron bursting -- this requires preventing premature activation, inducing motor neuron activation, and finally limiting the length of activation. Several groups of neurons may contribute to motor neuron activation. First, Hasegawa et al. (2016) identified two putative excitatory commissural premotor interneurons that promote motor neuron excitation, named cholinergic lateral interneuron 1 and 2 (CLI1 and CLI2) [47]. Based on the morphology, these are different from eIN-1, eIN-2, eIN-3 described above. CLI1 fires just before the aCC motor neuron only during forward crawling, while CLI2 fires prior to aCC during both forward and backward locomotion. Second, a large group of ~ 25 lateral locomotor neurons (LLNs) may provide excitatory input to motor neurons. LLNs show rhythmic activity during locomotion, and optogenetic activation results in muscle contraction, indicating they directly or indirectly excite motor neurons [72] (Fig. 4a). Although both CLIs and LLNs are likely to promote motor neuron excitation, there are many open questions: do LLNs directly connect to motor neurons? What is their neurotransmitter? What is the phase relationship between LLNs, CLIs and eINs? Do LLNs or CLIs synapse with all or a subset of motor neurons? An important step would be to identify LLNs and CLIs in the TEM volume so their pre- and post-synaptic partners could be identified.

Nothing is yet known about what prevents premature motor neuron activation (it could be absence of premotor excitation or presence of inhibition). In contrast, we have a much better idea of how motor neuron bursting is terminated. It appears to involve recruitment of inhibitory input, rather than cessation of excitatory drive. The Nose lab identified a group of ~ 20 glutamatergic inhibitory premotor neurons, known as Loopers or Period-positive Median Segmental Interneurons (PMSIs), which fire rhythmically with a short phase delay compared to motor neuron firing, and they promote efficient (fast) locomotion by limiting the length of motor neuron activation [64]. Direct inhibitory inputs from Loopers onto motor neurons were shown using GFP Reconstitution Across Synaptic Partners [64] and confirmed by electrophysiological recordings of inhibitory postsynaptic currents in two different motor neurons (RP2 and RP3) [73]. TEM reconstruction of a few looper neurons have shown that some are direct premotor neurons (A02b and A02m), whereas some (A02a, A02l) receive direct inputs from proprioceptors and are presynaptic to the GABAergic premotor neurons A27j and A31k (Fig. 4b). Since A27j and A31k neurons have not been examined at a functional or behavioral level, it is unknown if they have rhythmic firing pattern and are indeed involved in silencing the motor neurons during crawling. It is attractive to propose that some subsets of Loopers are mediators of the previously hypothesized “mission accomplished” signal [50] that promotes termination of motor neuron activity, which is required for rapid muscle contraction waves. In the future, it will be important to identify the Looper circuit partners in the TEM reconstruction to produce models of their role in regulating motor neuron bursting. It will also be important to develop more specific Gal4 or LexA lines that target subsets of these relatively large populations of interneurons. Lastly, it remains to be seen whether additional premotor neurons contribute to terminating motor neuron bursting.

### Intersegmental coordination: a feed-forward motif drives waves of motor activity

One of the fascinating features of locomotion across segmented or limbed metazoans is intersegmental coordination, by which the recruitment pattern of axial muscles or limbs stays proportional regardless of the pace of the movement cycle. Intersegmental coordination has been observed in a wide range of vertebrate and invertebrates during behavior in intact animals such as *Drosophila*, caterpillars, cockroaches, leeches, cats, and humans as well as in isolated brain preparations generating fictive motor patterns, including crustaceans, caterpillars, dogfish, and lampreys [22, 74–81]. These type of locomotory patterns are called phase constant, which means that the interval between segmental contractions scales linearly with the cycle period [32]. Just as the intrasegmental phase relationship between interneurons and motor neurons is observed in fictive preparations, so too is the intersegmental phase relationship between motor neurons, indicating that circuit mechanisms for both are located within the VNC and are not dependent on sensory feedback [32]. We note, however, that the duration of forward or backward waves in fictive preparations are ~ 10 times longer than in intact crawling larva [32]. This reduction in wave propagation speed in fictive preparations is likely due to lack of the “mission accomplished” signal from sensory proprioceptors.

New optogenetic and anatomical tools have made it possible to make progress on a cellular and circuit level description of how phase constant intersegmental coordination occurs during larval locomotion. A recent study from the Nose lab has discovered a feed-forward inhibitory motif that promotes intersegmental coordination. This motif, which spans two adjacent segments, is composed of a cholinergic excitatory premotor neuron (A27h) and a pre-premotor GABAergic dorsolateral interneuron (GDL). GDL is rhythmically active just prior and concurrent with motor neurons, and silencing it significantly slows forward locomotion. TEM connectome analysis shows that A27h receives input from the GDL in the same segment, but provides input to GDL in the next anterior segment. This suggests a feed-forward circuit where A27h activates motor neurons in one segment, as well as preventing premature A27h activation in the next most anterior segment (via activating the GDL inhibitory neuron) [46] (Fig. 4c). In addition, GDL receives direct input from somatosensory neurons [46], which could help tune the length of the intersegmental delay.

### Forward and backward locomotion recruit distinct premotor interneurons

The excitatory premotor neuron A27h described in the previous section is interesting because it was the first neuron shown to be rhythmically active during forward but not backward locomotion [46]. This makes sense in light of the feedforward circuit it uses to modulate the timing of forward peristaltic waves, which would not function in the reverse direction to promote coordinated backward locomotion (Fig. 4c). More recently, a segmentally repeated “command-like” neuron called Wave (A02o) has been discovered which upon optogenetic activation in anterior segments, triggers backward crawling [82]. Calcium imaging of Wave neurons in isolated brains indicates that they are not recruited in forward or backward locomotion [82]. Interestingly, Wave neurons receive synaptic inputs from class III/IV md neurons, indicating that they relay nociceptive sensory information to the motor circuits. It will be of a great interest to examine how these nociceptive signals are being translated at the level of premotor and motor neurons. Despite progress, many important questions remain. Is the pattern of muscle contractions different in forward and backward locomotion? Are any motor neurons differentially active in forward and backward locomotion? Finally, very few premotor neurons have been analyzed for activity or function: how many are differentially active in forward and backward locomotion?

### Left-right symmetric motor output

Not only is the precise timing of intrasegmental or intersegmental motor activity important for locomotion, it is also essential that there is left and right synchronous and symmetric motor output [16]. In a screen for neuronal activation phenotypes that disrupted larval locomotion, a pair of Gal4 lines were identified that had the same phenotype and showed overlapping expression in just five interneurons -- a subset of the interneurons expressing the Even-skipped (Eve) transcription factor called the Eve Lateral (EL) neurons. These neurons are conserved in flies, fish and mouse as being excitatory, contralateral ascending interneurons [83]. When five of these EL neurons were activated (or silenced) it resulted in a slow locomotor phenotype where left and right muscle groups continued to contract synchronously (the CPG driving motor output was unaffected) but muscle contraction amplitudes were uncoordinated and the larvae showed “wavy” body posture [16]. Multicolor flip out (MCFO) was done to identify the precise morphology of these neurons, which allowed them to be identified in the TEM reconstruction, and their circuitry revealed. Interestingly, the EL neurons are at the core of a sensorimotor circuit, with proprioceptive input (directly or indirectly via three Jaam interneurons), and downstream motor output (directly or indirectly via three Saaghi premotor neurons) [16]. It was proposed that slight differences in left/right muscle length produce unequal activation of EL neurons, which then activate premotor/motor neurons to restore left/right symmetric muscle lengths. It is currently unknown whether the Eve transcription factor is required for any aspect of this connectivity or function; similarly, whether mutants in the vertebrate Evx transcription factor produce similar phenotypes awaits more precise behavioral analysis than has been done to date.

### The role of sensory input in larval locomotion

Although this review is focused on forward and backward locomotion, in this section we add mention of a neural circuit driving larval escape behavior. Larval defense against attack from parasitoid wasps requires Class IV md neurons [54, 84]. The first use of optogenetics in Drosophila larvae drove channelrhodopsin in Class IV md neurons to induce the same rolling escape response as being attacked by a predatory wasp. Strikingly, Ohyama et al. showed that synergistic activation of Class IV md neurons along with mechanosensory chordotonal neurons increases the chance of rolling behavior [85]. The Zlatic and Cardona labs used TEM connectomics to identify neural circuits downstream of the Class IV md neurons as well as chordotonal neurons, and in concert with modern optogenetic tools, they characterized the circuit mechanism for larval escape [85, 86] (Fig. 4d). They showed that the md and chordotonal sensory neurons preferentially provide input to different Basin neurons within the same segment; the Basins then project to the A05q and A23g interneurons; and finally A05q and A23g target the Goro command neuron. In addition, the Basin neurons can also activate Goro via an indirect pathway using A00c ascending neurons that then connect to central brain descending neurons innervating Goro*.* It remains unclear how Goro triggers the motor program involved in the rolling escape behavior; in fact, the pattern of motor neuron and muscle activity during rolling escape behavior remains to be determined.

## Conclusions

The neural circuits controlling Drosophila larval locomotion are being characterized with great rapidity; however, much is still required before we can fully understand larval crawling. Are all muscles used for all translocating locomotor behaviors (forward, backward, rolling)? Are all motor neurons used, including Ib and Is motor neurons? How many premotor neurons exist, and which are used for each translocating larval behavior? How many excitatory premotor neurons are required to make a motor neuron burst? Are any premotor or motor neurons specifically used in a single behavior? Last but not least, how is sensory information generated in the periphery and processed in the VNC to allow smooth locomotor behavior? Identification of the remaining motor and premotor neurons in the TEM data set, along with functional validation of their synaptic connections will help answer these questions and reveal how an ensemble of premotor interneurons sculpt the stereotypic muscle contraction pattern during forward and backward locomotion.

## Abbreviations

CLI: cholinergic lateral interneuron
CNS: central nervous system
CPGs: central pattern generators
da: dendritic arborization
EL: Eve Lateral
GDL: GABAergic dorsolateral
L: longitudinal
LLN: lateral locomotor neuron
md: Multidendritic
PMSI: Period-positive Median Segmental Interneuron
SEZ: subesophageal zone
T: transverse
TEM: transmission electron microscopy
VNC: ventral nerve cord
